# Supplementary material for: 5-aminosalicylic acid suppresses osteoarthritis through the OSCAR-PPARγ axis
Source: Nat Commun. 2024 Feb 3;15:1024. doi: 10.1038/s41467-024-45174-6 (PMC10838344; doi:10.1038/s41467-024-45174-6)
Supplement: Supplementary file 2 — Supplementary Information [file 41467_2024_45174_MOESM2_ESM.pdf]

Supplementary Information

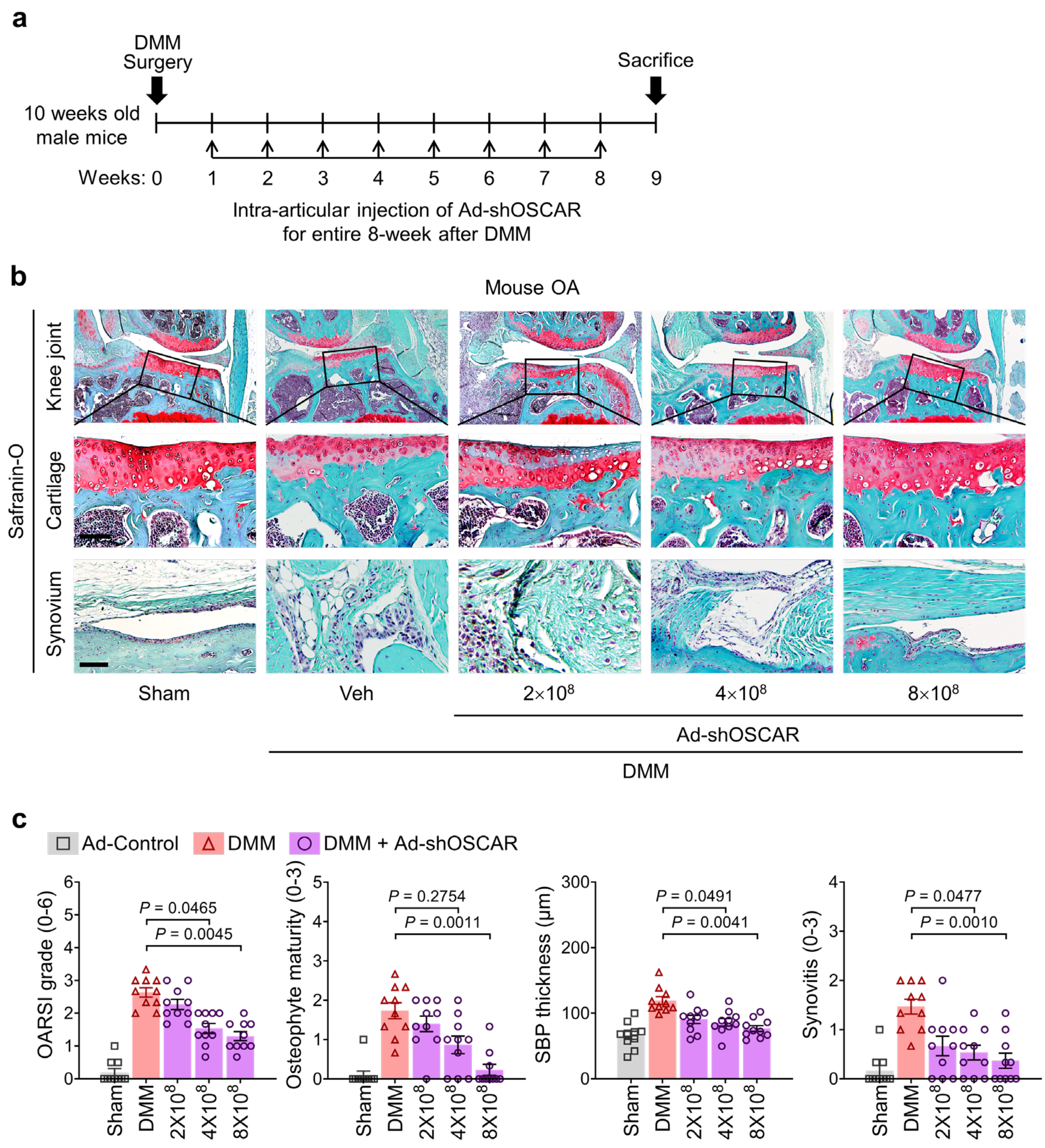

Supplementary Fig. 1. Osteoarthritis can be suppressed by knocking down OSCAR.

a Schematic depiction of Ad-shOSCAR treatment of mice that underwent DMM surgery to induce OA. **b**, **c** The knee joints of C57BL/6-J mice were subjected to sham operation or DMM surgery. The joints were also injected IA with Ad-shOSCAR or Adeno-control (Ad-C) (MOIs

of 2×10^8, 4×10^8, and 8×10^8) once a week for 8 weeks starting 1 week after surgery. The mice were sacrificed 9 weeks after the operation (n = 10 mice per group). The knee joints were sectioned and stained with Safranin-O staining and fast green counterstaining. **b** shows representative images. **c** shows the effect of Ad-shOSCAR on the following OA variables: OARSI grade, osteophyte maturity, SBP thickness, and synovitis. The OARSI grade, synovitis and osteophyte maturity data are shown as means ± 95% confidence intervals (CI). Differences between groups were determined with Kruskal-Wallis test followed by Mann–Whitney U test. Means ± s.e.m. with two-tailed t-test for SBP thickness. Exact P values can be found in the accompanying Source Data. Scale bars, 25 μm.

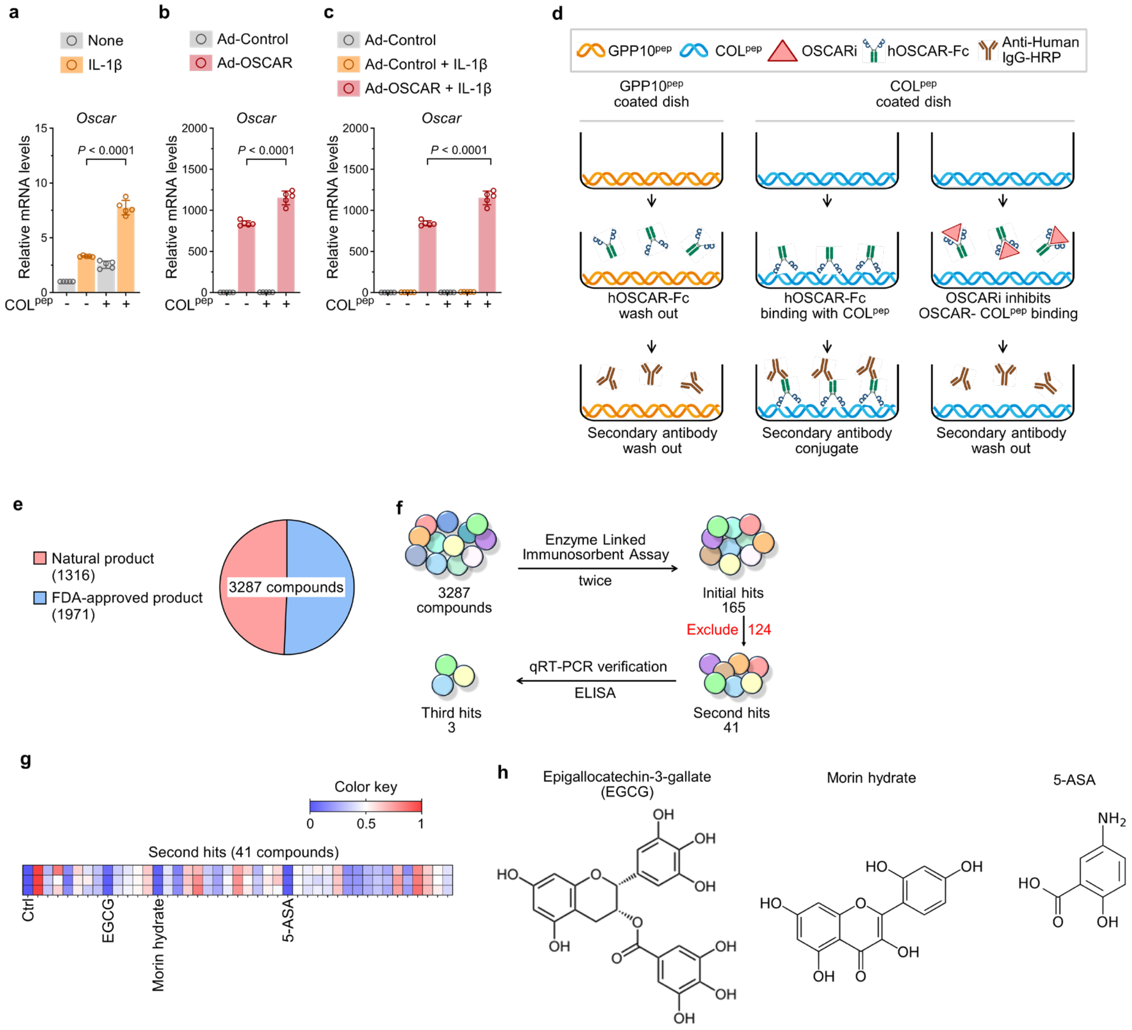

Supplementary Fig. 2. Screening for DMOAD candidate therapeutics by using a high-throughput format.

a Readouts of the binding of COL^{pep} to OSCAR on the chondrocyte cell surface. This binding event leads to upregulation of OSCAR transcription[15]. (Left) Primary (uninfected) chondrocytes. Culture on immobilized COL^{pep} alone, or with IL-β alone, slightly increases native OSCAR expression. Both stimuli markedly elevate native OSCAR expression. (Right) Ad-OSCAR-infected chondrocytes: adenovirus-derived/native OSCAR expression is elevated by culture on COL^{pep} alone and further augmented when IL-1β is also present. The readout is qRT-PCR-determined OSCAR mRNA levels or immunoblotting-determined OSCAR protein levels. **d**, **e** Schematic depiction of the ELISA screening system that was used to detect small-

molecule inhibitors of the binding of OSCAR to collagen. Small compounds from two different libraries (**e**) were mixed with purified human Fc-OSCAR protein and added to ELISA plates that had been pre-coated with OSCAR-binding collagen peptide (COL^{pep}). Horseradish peroxidase-conjugated anti-human IgG served as the secondary antibody. The readout was OD450. **f** Schematic depiction of the screening process. ELISA was used to find first hits. Candidates that were previously reported (n = 124, Supplementary Table 1) were excluded, leaving 41 candidates. The candidates that competed most strongly with COL^{pep} (n = 3) were selected for verification dose-ELISA. **g** ELISA heatmap of the 41 second-hit OSCAR inhibitors. Control, wells coated with negative control collagen mimetic peptide GPP10. +Control, wells coated with COL^{pep} and treated with OSCAR-Fc alone. **h** Structure of the three strongly competing candidates, namely, epigallocatechin-3-gallate, morin hydrate, and 5-ASA. *P*-values were determined by one-way ANOVA followed by Tukey's multiple comparison's test. The schematic illustration in the figure created with BioRender.com.

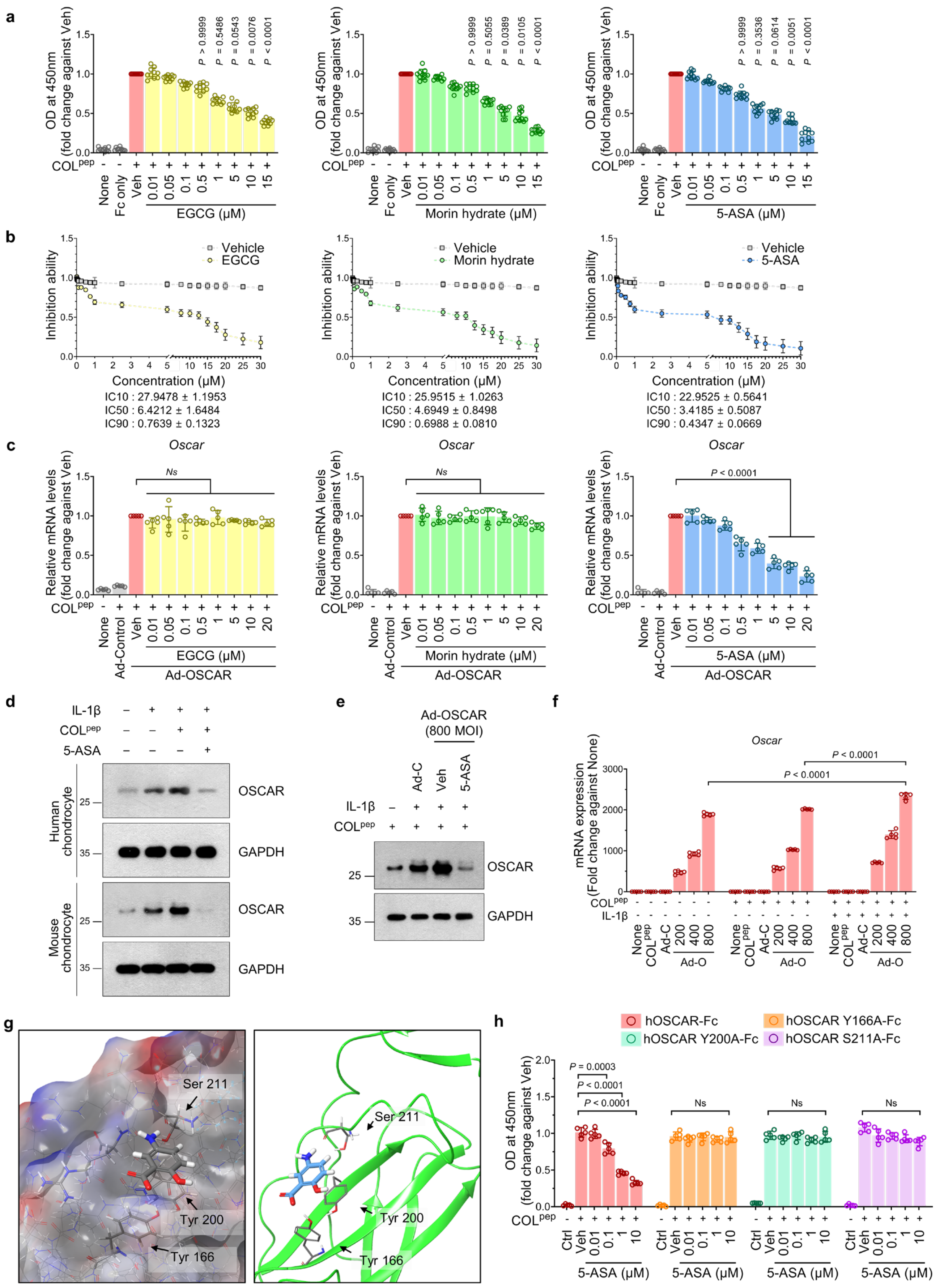

Supplementary Fig. 3. Identifying 5-ASA as an OSCAR antagonist.

a Verification ELISA. COL^{pep}-coated plates were incubated with hOSCAR-Fc mixed with increasing concentrations of each candidate to determine whether the three candidates

could compete with COL^{pep} for OSCAR. **b** IC10, IC50 and IC90 values of EGCG, Morin hydrate and 5-ASA. **c-e** In vitro experiments to determine whether the three candidates could compete with COL^{pep} for OSCAR on the surface of primary murine chondrocytes. The binding of COL^{pep} to OSCAR on chondrocytes promotes OSCAR expression[14]. **c** Chondrocytes were infected for 2h with Ad-OSCAR (MOI 800) on COL^{pep}-coated or uncoated plates without IL-1β, treated for 48h with each of the three candidates. **d-f** Chondrocytes were cultured for 48h on COL^{pep}-coated or uncoated plates without or with IL-1β, and then treated with 5-ASA for 48h (**d**), or infected for 2h with indicated MOI of Ad-OSCAR (200, 400 or 800 MOI) (**e**, **f**). The indicated dose of each candidate (**b**) or 10 μM 5-ASA (**d**, **e**) was then added. Vehicle (Veh) was DMSO. OSCAR mRNA (**c**) or protein (**d, e**) was then measured by RT-PCR and western blotting, respectively. Representative western blots are shown. **g** Induced-fit docking analysis to determine the key OSCAR residues that bind to 5-ASA. Three different binding site poses of 5-ASA with hOSCAR-Fc were predicted and shown. The locations of the Tyr166, Tyr200 and Ser211 residues are indicated (arrows). The residues comprising the binding cavity are depicted with a stick model inside an electrostatic transparent surface potential mapping on OSCAR (left). The interaction pose is also displayed with a ribbon model (right). Red colors represent for negative charges and blue for positive, respectively. 5-ASA is drawn with thick sticks. **h** Effect of mutating the Tyr166, Tyr200 and Ser211 residues to arginine by site-directed mutagenesis on 5-ASA competition with COL^{pep} for OSCAR. ELISA was conducted on COL^{pep} coated plates with wildtype hOSCAR-Fc, hOSCAR Y166A-Fc, hOSCAR Y200A-Fc, or hOSCAR S211A-Fc in the presence or absence of 5-ASA. Optical density at 450 nm (OD 450) was determined. *P*-values were determined by Kruskal-Wallis test followed by Dunn's multiple comparisons test (**a**), or one-way ANOVA followed by Tukey's multiple comparison test (**b**, **c**), or two-way ANOVA followed by Sidak's multiple comparisons test (**f**, **h**).

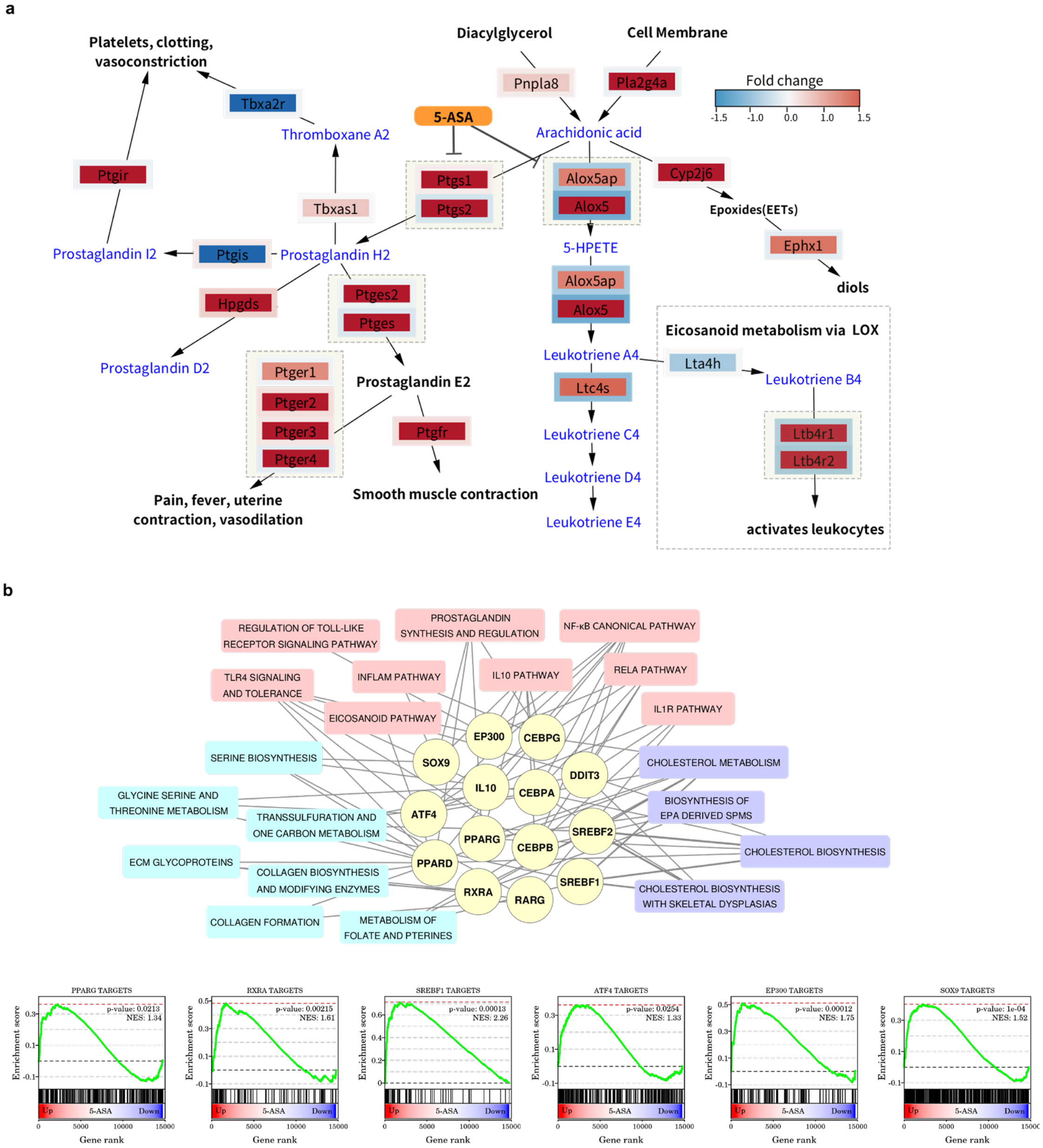

Supplementary Fig. 4. 5-ASA may reduce inflammation in OA by downregulating the eicosanoid pathway.

a (left) The protein-protein interaction network that is up/downregulated by OSCAR overexpression but then flipped by 5-ASA. The proteins encoded by the flipped DEGs are colored according to the degree of log2-scale fold change caused by OSCAR overexpression (center of boxes) and 5-ASA (border). Blue indicates downregulation while pink/red indicates upregulation. Thus, when a protein/DEG is up-regulated by OSCAR overexpression but this is

flipped by 5-ASA, the box is pink/red in the center and blue at its border. 5-ASA particularly strongly downregulates Ptgs2 and Alox5 (yellow box linked with blocked arrows). (right) The relationship between OSCAR overexpression and the genes belonging to the eicosanoid-related pathways in MsigDB. The blue-pink/red color scheme used in the protein network is also employed to denote the effect of 5-ASA treatment (border of circles) on OSCAR-regulated DEGs (center). The DEGs that are most strongly affected by OSCAR are connected to OSCAR by red lines. The next most strongly affected DEGs are connected with orange lines. **b** The normalized enrichment score (NES) for downstream genes of each TF was calculated by gene-set enrichment analysis (GSEA).

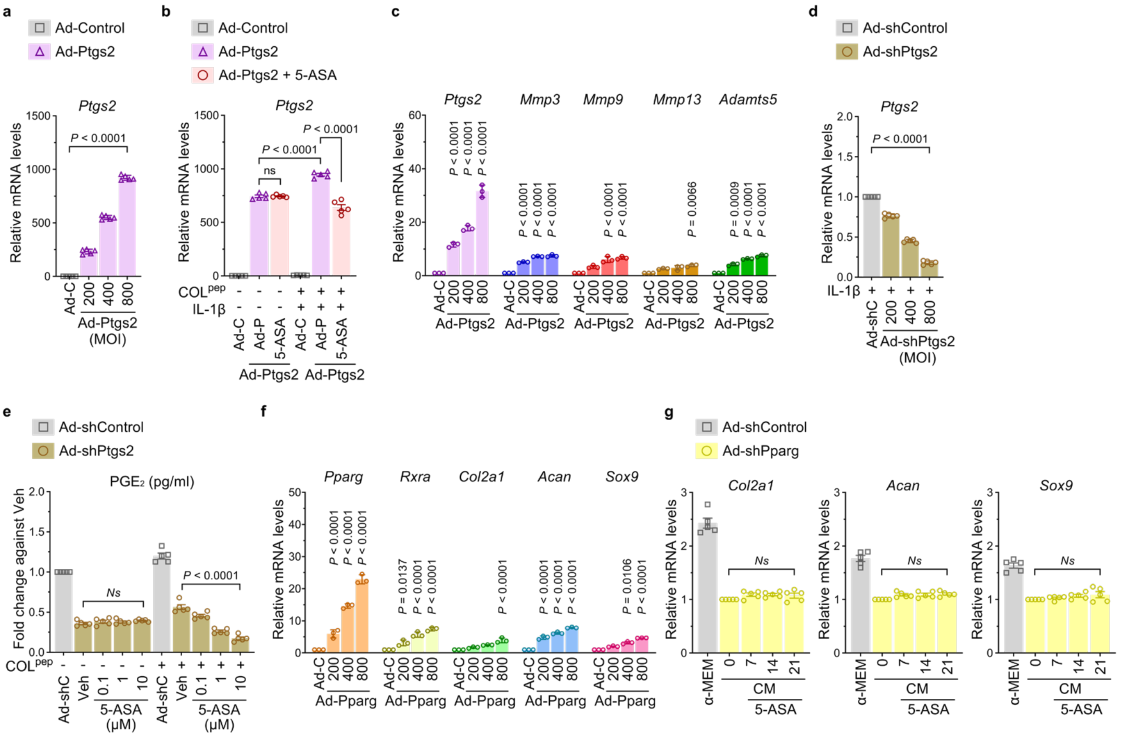

Supplementary Fig. 5. 5-ASA binding to OSCAR suppresses COL^{pep}-induced COX-2 expression in chondrocytes by activating PPARγ.

a Primary murine chondrocytes were infected with Ad-Control (Ad-C; 800 MOI) or the indicated Ad-Ptgs2 MOI for 2 h and *Ptgs2* mRNA was measured with qRT-PCR. **b** Primary chondrocytes were infected with Ad-Control (Ad-C; 400 MOI) or Ad-Ptgs2 (400 MOI) for 2 h, treated with 3 µg/ml COL^{pep} + 10 ng/ml IL-1β with or without 10 µM 5-ASA for 48 h, and *Ptgs2* mRNA was measured with qRT-PCR. **c** Primary chondrocytes were infected with Ad-Control (Ad-C; 800 MOI) or the indicated Ad-Ptgs2 MOI for 2 h and *Ptgs2*, *Mmp3*, *Mmp9*, *Mmp13* and *Adamts5* mRNA was measured with qRT-PCR. **d** Primary chondrocytes were infected with Ad-shControl (Ad-shC; 800 MOI) or the indicated Ad-shPtgs2 MOI for 2 h and *Ptgs2* mRNA was measured with qRT-PCR. **e** Primary chondrocytes were infected with Ad-shControl (Ad-shC; 800 MOI) or Ad-shPtgs2 (800 MOI) for 2 h and treated with 3 µg/ml COL^{pep} + 10 ng/ml IL-1β with or without the indicated 5-ASA concentration for 48 h. ELISA was used to measure the PGE2 that was

released into the supernatant. **f** Primary chondrocytes were infected with Ad-Control (Ad-C; 800 MOI) or the indicated Ad-Pparg MOI for 2 h and *Pparg*, *Rxra*, *Col2a1*, *Acan* and *SOX9* mRNA was measured with qRT-PCR. **g** Murine bone marrow-derived MSCs were infected with Ad-shControl (Ad-shC; 800 MOI) or Ad-shPparg (800 MOI) for 2 h and cultured in chondrogenic medium (CM) with and without 5-ASA for 7, 14 and 21 days, and qRT-PCR was conducted to determine *Col2a1*, *Acan* and *SOX9*. The data are shown as means ± s.e.m. *P*-values were obtained by one-way ANOVA followed by Tukey's multiple comparisons test (**a**, **d**, **g**), or two-way ANOVA followed by Sidak's multiple comparisons test (**b**, **c**, **e**, **f**).

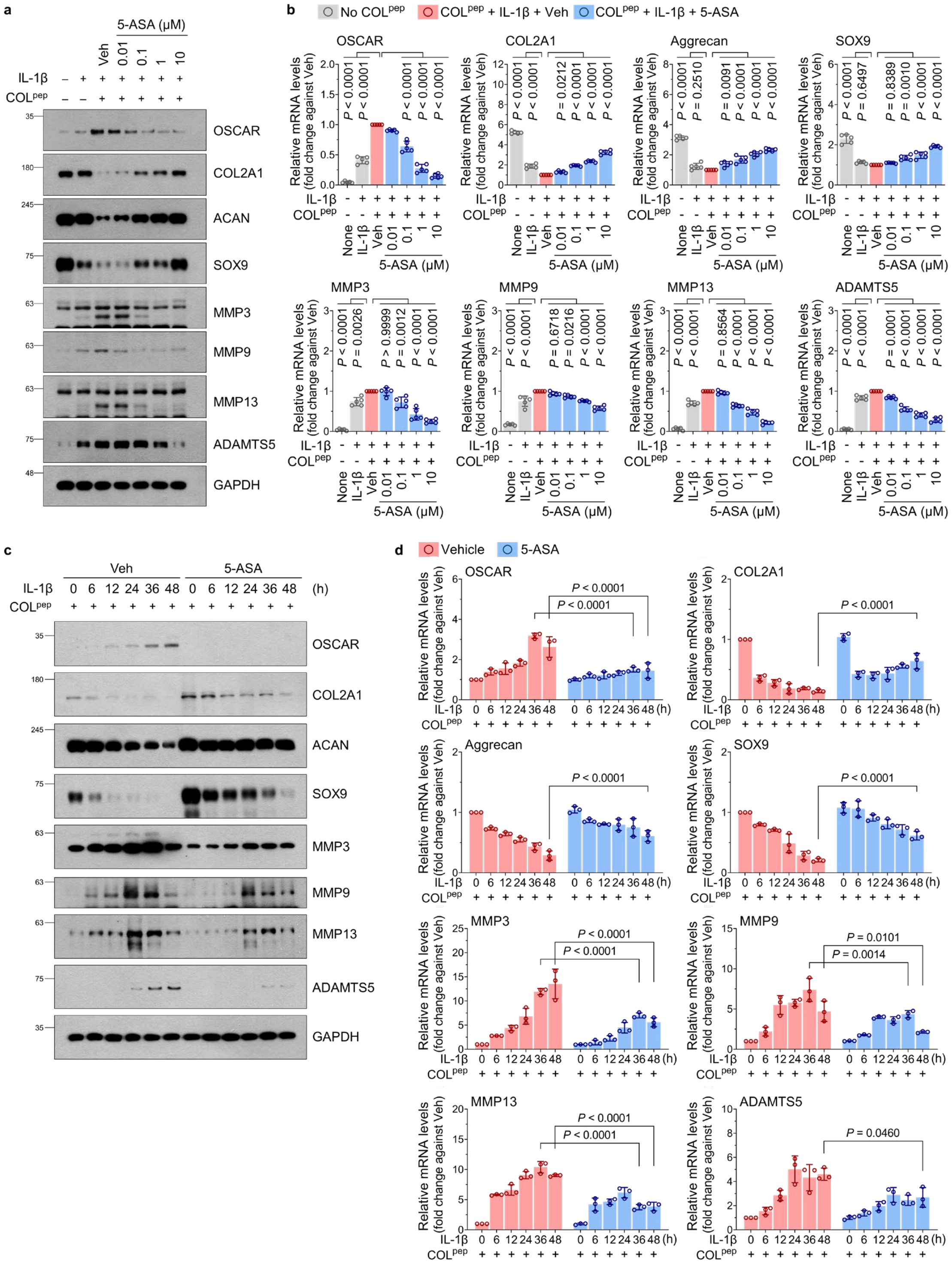

Supplementary Fig. 6. 5-ASA targets catabolism and anabolism in primary OSCAR-expressing chondrocytes.

Primary mouse chondrocytes were cultured on dishes with/without 10 ng/ml IL-1β and

with/without 5μM 5-ASA for 48 h (*n* = 5 independent primary chondrocyte cultures). **a**, **b** The cultures were on dishes coated with 2 μg/ml GPP10 control peptide or COL^{pep}. **c**, **d** The cultures were on uncoated dishes. In **a**, **c**, Western blotting was conducted with OSCAR, COL2A1, ACAN, SOX9, MMP3, MMP9, MMP13 and ADAMTS5 antibodies. In **b**, **d**, qRT-PCR was conducted for OSCAR, COL2A1, ACAN, SOX9, MMP3, MMP9, MMP13, and ADAMTS5 mRNA. The data are shown as means ± s.e.m. *P*-values were obtained by one-way ANOVA followed by Tukey's multiple comparisons test (**b**), or two-way ANOVA followed by Sidak's multiple comparisons test (**d**).

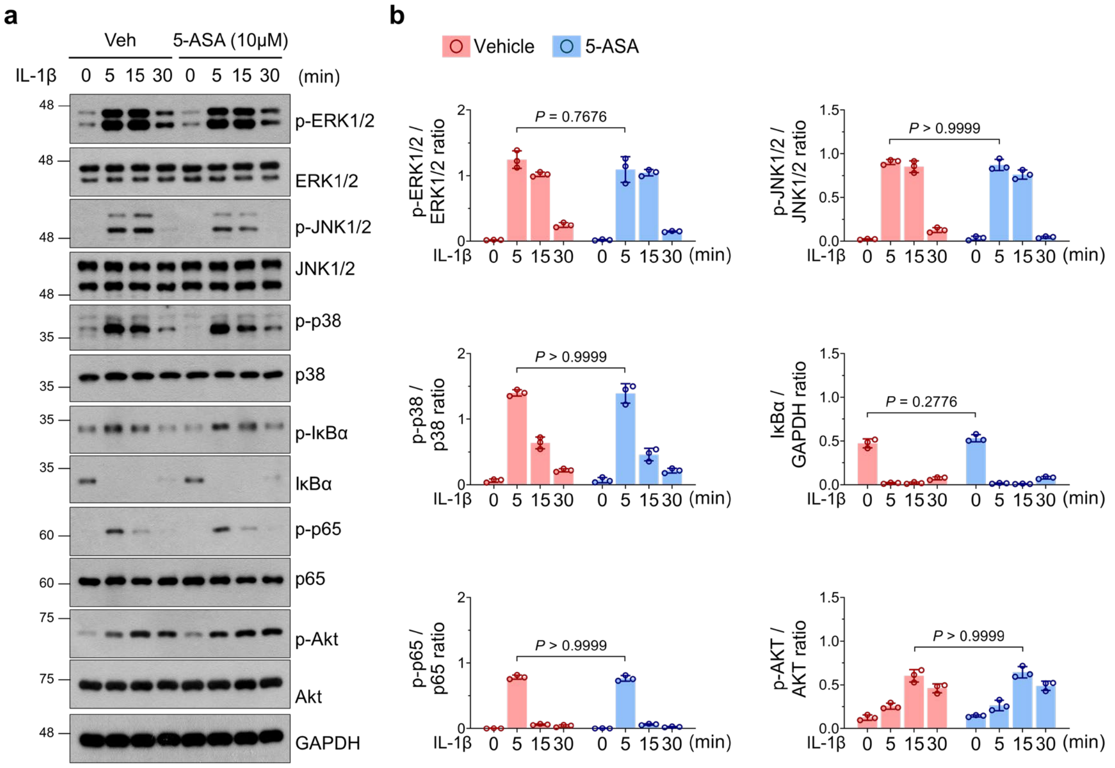

Supplementary Fig. 7. 5-ASA does not alter IL-1β-induced MAPK and NF-κB pathways in chondrocytes.

ICR murine chondrocytes were serum-starved for 6 hours and stimulated with 10 ng/ml IL-1β in the presence of 10 μM 5-ASA or DMSO for the indicated times. Western blot analysis was conducted with the indicated antibodies (**a**) and the results were then quantified (**b**). Three independent experiments were conducted. These data are shown as means ± s.e.m. Two-way ANOVA was conducted followed by Sidak's multiple comparisons test.

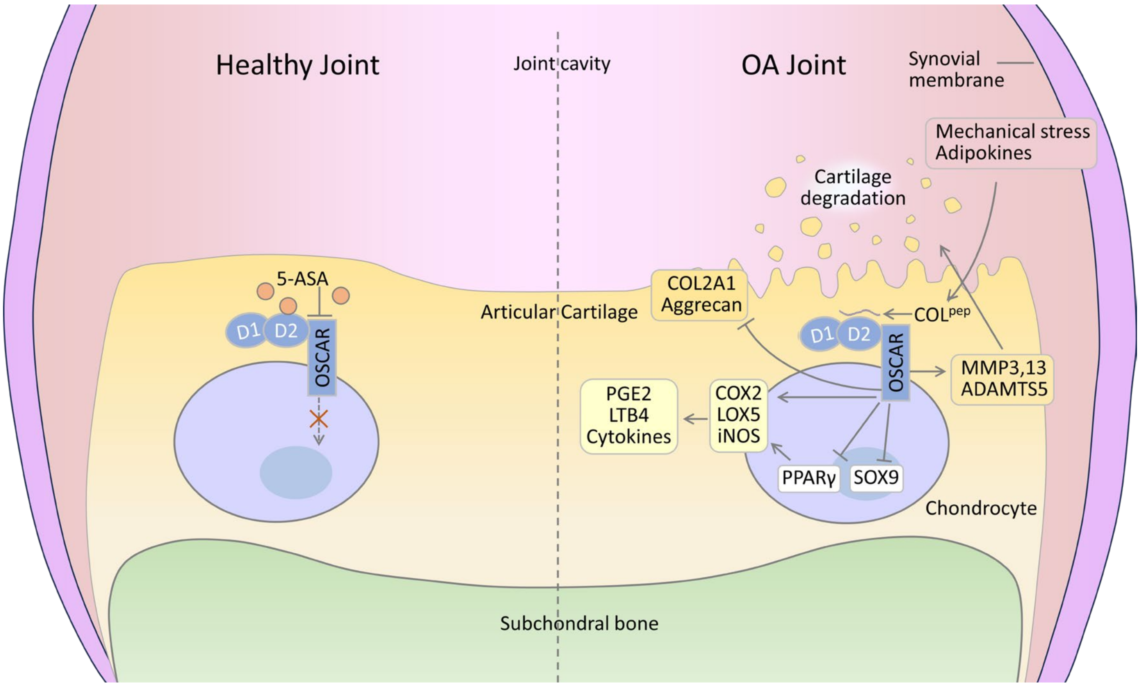

Supplementary Fig. 8. Proposed model by which 5-ASA could act as a therapeutic agent during OA pathogenesis.

OSCAR-induced OA pathogenesis is negatively regulated by 5-ASA. Healthy cartilage can be maintained by 5-ASA, which inhibits OSCAR. The schematic illustration in the figure created with BioRender.com.

Figure 1a

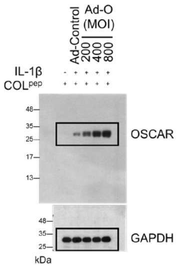

Figure 3a

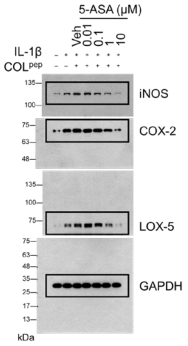

Figure 3f

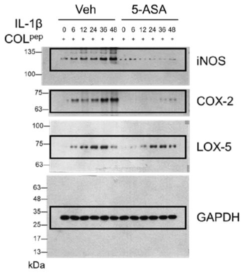

Supplementary Figure 3d

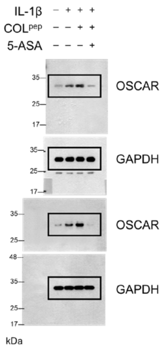

Supplementary Figure 3e

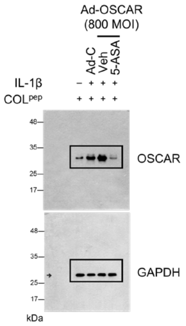

Supplementary Figure 6a

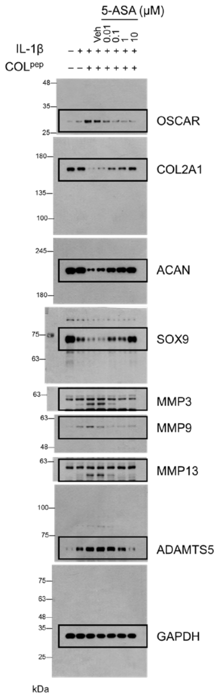

Supplementary Figure 6c

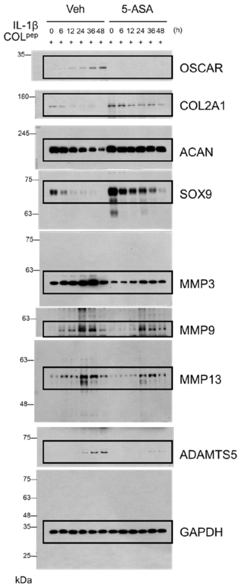

Supplementary Figure 7a

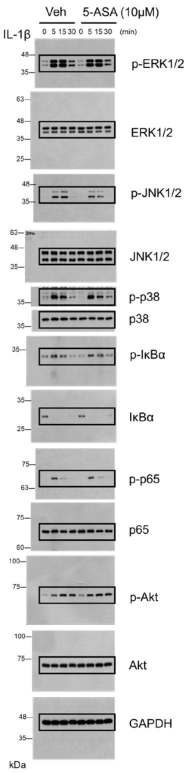

Supplementary Fig. 9. Uncropped scans of all blots.

Uncropped scans with size marker indications.
