# Supplementary material for: 5-aminosalicylic acid suppresses osteoarthritis through the OSCAR-PPARγ axis
Source: Nat Commun. 2024 Feb 3;15:1024. doi: 10.1038/s41467-024-45174-6 (PMC10838344; doi:10.1038/s41467-024-45174-6)
Supplement: Supplementary file 4 — Reporting Summary [file 41467_2024_45174_MOESM4_ESM.pdf]

nature portfolio

Corresponding author(s): Soo Young Lee

Last updated by author(s): Jan 2, 2024

Reporting Summary

Nature Portfolio wishes to improve the reproducibility of the work that we publish. This form provides structure for consistency and transparency in reporting. For further information on Nature Portfolio policies, see our Editorial Policies and the Editorial Policy Checklist.

Statistics

For all statistical analyses, confirm that the following items are present in the figure legend, table legend, main text, or Methods section.

n/a	Confirmed	
☐	☒	The exact sample size (*n*) for each experimental group/condition, given as a discrete number and unit of measurement
☐	☒	A statement on whether measurements were taken from distinct samples or whether the same sample was measured repeatedly
☐	☒	The statistical test(s) used AND whether they are one- or two-sided<br>*Only common tests should be described solely by name; describe more complex techniques in the Methods section.*
☐	☒	A description of all covariates tested
☐	☒	A description of any assumptions or corrections, such as tests of normality and adjustment for multiple comparisons
☐	☒	A full description of the statistical parameters including central tendency (e.g. means) or other basic estimates (e.g. regression coefficient) AND variation (e.g. standard deviation) or associated estimates of uncertainty (e.g. confidence intervals)
☐	☒	For null hypothesis testing, the test statistic (e.g. *F*, *t*, *r*) with confidence intervals, effect sizes, degrees of freedom and *P* value noted<br>*Give P values as exact values whenever suitable.*
☒	☐	For Bayesian analysis, information on the choice of priors and Markov chain Monte Carlo settings
☒	☐	For hierarchical and complex designs, identification of the appropriate level for tests and full reporting of outcomes
☒	☐	Estimates of effect sizes (e.g. Cohen's *d*, Pearson's *r*), indicating how they were calculated

Our web collection on statistics for biologists contains articles on many of the points above.

Software and code

Policy information about availability of computer code

Data collection	Histological and immunohistochemical staining images Image were acquired using the Olympus DP72 (version 2.1) camera. For immunofluorescence staining, the stained cells were Imarged using a Zeiss 880 Airyscan and OsteoMeasure XP version 2.0.0.2. For RNA-seq analysis, all procedures were performed by Macrogen using an Illumina NovaSeq 6000 system. Networks were visualised by Cytoscape v3.8 software. Micro-CT data were scanned by Skyscan1176 and the raw micro-CT data were translated into 2-dimensional cross-sectional gray-scale images slices by using Nrecon (Brucker micro-CT), after which the following structural variables of the trabecular and cortical bones were measured by CT Analyzer (CT-AN ver.1.10.9.0). Quantification of the western blot was performed using the program Media Cybernetics Image-pro plus 4.5 and ImageJ.<br>For qRT-PCR analysis, all procedures were performed by StepOne™ Software, version 2.3. Graphpad Prism 8.4.3 was used for data analysis and collection. A schematic scheme was created using BioRender.com.
Data analysis	Sclerosis and articular cartilage destruction were identified by safranin-O staining and measured with OsteoMeasureXP (ver.2.0.0.2 OsteoMetrics, Inc., Atlanta, GA, USA), Image-pro plus (v4.5, Media Cybernetics, Inc., Rockville, USA), Adobe photoshop (v9.0, San Jose, CA, USA), and an Olympus DP72 charge-coupled device camera (v2.1, Olympus Corporation, Tokyo, Japan).<br>Protein band intensity was quantified by densitometric analysis using ImageJ (v1.53e). To quantitatively analyze mRNA transcript levels, cDNA was amplified by qRT-PCR on StepOne™ Plus Real-Time PCR system Software, v2.3 (Applied Biosystems).<br>For Induced-Fit Docking analysis, all the docking and scoring calculations were performed using the Schrödinger software suite (Maestro, version 11.8.012). The SDF file of 5-ASA was acquired from the PubChem database.<br>For RNA sequencing analysis data, the PPI network was collected from STRING database v.11.0. Networks were visualized by Cytoscape v.3.8 software, STAR aligner (version2.7.1a), and normalized TPM (Transcripts Per Kilobase Million) values of each gene were calculated using RSEM (version 1.2.17) with Gencode v22 annotation. Raw read counts were obtained by RSEM v.1.3.1.<br>Micro CT analysis data was using Skyscan 1176, and CT Analyzer.

Statistical analyses were performed using GraphPad Prism (v8.4.3, San Diego, CA, USA) or IBM SPSS Statistics 25. All the graphs and heatmaps of log2(fold change) were plotted using GraphPad Prism v8.4.3).

For manuscripts utilizing custom algorithms or software that are central to the research but not yet described in published literature, software must be made available to editors and reviewers. We strongly encourage code deposition in a community repository (e.g. GitHub). See the Nature Portfolio guidelines for submitting code & software for further information.

Data

Policy information about availability of data

All manuscripts must include a data availability statement. This statement should provide the following information, where applicable:

- Accession codes, unique identifiers, or web links for publicly available datasets
- A description of any restrictions on data availability
- For clinical datasets or third party data, please ensure that the statement adheres to our policy

The data that support the findings of this study are available within the article and its Supplementary Information files or from the corresponding author on reasonable request. Source data are provided with this paper. RNA-seq data of the Ad-OSCAR-infected and 5-ASA-treated chondrocytes can be obtained from Gene Expression Omnibus (GEO) under accession number GSE207056. The following figures have associated raw data: Figures 1b, 1f, 1i, 3b, 3d-e, 3g, 4a-c, 4e-i, 5c, 5e, 5h, 6c and 7a-f; Supplementary Figures 1c, 2a–c, 2g, 3a-c, 3f, 3h, 5a-g, 6b, 6d and 7b. For gel Source data, see Supplementary Data 1.

Research involving human participants, their data, or biological material

Policy information about studies with human participants or human data. See also policy information about sex, gender (identity/presentation), and sexual orientation and race, ethnicity and racism.

Reporting on sex and gender	10 human specimens, comprising 7 female and 3 male patients, underwent total knee replacement surgery.
Reporting on race, ethnicity, or other socially relevant groupings	*Please specify the socially constructed or socially relevant categorization variable(s) used in your manuscript and explain why they were used. Please note that such variables should not be used as proxies for other socially constructed/relevant variables (for example, race or ethnicity should not be used as a proxy for socioeconomic status).*<br>*Provide clear definitions of the relevant terms used, how they were provided (by the participants/respondents, the researchers, or third parties), and the method(s) used to classify people into the different categories (e.g. self-report, census or administrative data, social media data, etc.)*<br>*Please provide details about how you controlled for confounding variables in your analyses.*
Population characteristics	International Cartilage Repair Society (ICRS) grade 4 cartilage tissues were obtained from patients with osteoarthritis aged from 63 to 78 years during total knee replacement surgery. The cartilage tissue offered by these patients were grouped by intact and damaged region for the following analysis. No patients with osteoarthritis had rheumatoid arthritis, metabolic diseases, or other inflammatory diseases at the time of surgery.
Recruitment	Patients undergoing surgery were recruited for offering cartilage on an 'as available' basis in Gangnam Severance Hospital. The protocol, including the use of cartilage, was approved by the Ethics Committee of Yonsei University Gangnam Severance Hospital, Seoul, Korea. All participating patients provided signed informed consent. There exists the potential for self-selection bias or other forms of bias, which could inadvertently skew the results contrary to our intentions.
Ethics oversight	Human subjects research was performed according to the Institutional Review Boards at Yonsei University Gangnam Severance Hospital. The institutional review board of Yonsei University (Protocol No: IRB 3-2018-0251), Gangnam Severance Hospital, South Korea approved the use of the articular cartilage. All participants provided written informed consent for the use of their tissues for research purposes.

Note that full information on the approval of the study protocol must also be provided in the manuscript.

Field-specific reporting

Please select the one below that is the best fit for your research. If you are not sure, read the appropriate sections before making your selection.

☒ Life sciences ☐ Behavioural & social sciences ☐ Ecological, evolutionary & environmental sciences

For a reference copy of the document with all sections, see nature.com/documents/nr-reporting-summary-flat.pdf

Life sciences study design

All studies must disclose on these points even when the disclosure is negative.

Sample size	Statistical methods were used to predetermined sample size in vitro and in vivo analyses. We designed in vivo experiments to ensure that minimum number of mice are used to obtain biologically significant results. Sample sizes were chosen based on previous utilizing these outcome measures (PMID: 32859940, 37893216). Sample sizes were deemed to be sufficient based on size of effects seen and reaching statistical significance based on the available samples. For in vivo osteoarthritis experiments, n≥10 was used and experiments were repeated twice. For experiments in which large biological difference was expected such as OARSI scoring of cartilage rescue from DMM-induced osteoarthritis , at least n=10 was utilized and experiments were repeated twice. For human data, at least n=10 was used for gene expression experiments due to large variability in human samples.

Data exclusions	No data was excluded from analyses.
Replication	All findings were replicated in a at least four independent experiments performed under identical conditions.
Randomization	For in vitro experiments, samples were randomly allocated to different experimental groups prior to treatment. Additionally, cultures were selected randomly for various treatments. To ensure the robustness and reproducibility of our results, the experiments were performed multiple times.<br>For mouse experiments, mice were randomly assigned by a technician blinded to the experimental design. Male mice were selected for the experiment in order to avoid concerns about hormonal effect in female mice. After the selection, mice for the DMM surgery, or intra-articular injection were allocated randomly without subjective judgement.
Blinding	Investigators were not blinded during sample collection for qPCR, western blotting or immunofluorescence experiments. However, these results of these assays are not subjective and provide quantitative data, removing the need for blinding. Only histological analysis required subjective grading of samples.Investigators were blinded to the cartilage destruction analysis, Subchondral bone plate thickness analysis, ratio of hyaline and calcified cartilage analysis of mice during histological analysis and scoring.

Reporting for specific materials, systems and methods

We require information from authors about some types of materials, experimental systems and methods used in many studies. Here, indicate whether each material, system or method listed is relevant to your study. If you are not sure if a list item applies to your research, read the appropriate section before selecting a response.

Materials & experimental systems

n/a	Involved in the study
☐	☒ Antibodies
☐	☒ Eukaryotic cell lines
☒	☐ Palaeontology and archaeology
☐	☒ Animals and other organisms
☒	☐ Clinical data
☒	☐ Dual use research of concern
☒	☐ Plants

Methods

n/a	Involved in the study
☒	☐ ChIP-seq
☒	☐ Flow cytometry
☒	☐ MRI-based neuroimaging

Antibodies

Antibodies used	Primary antibodies used for immunohistochemistry were as follows;<br>OSCAR (1:200; Santa Cruz Biotechnology; sc34235; Lot#E2115), OSCAR (1:200; Biorbyt; orb185679; Lot#BR5396), MMP3 (1:50; Abcam; ab53015; Lot#GR3228789-8), MMP13 (1:25; Abcam; ab51072; Lot#GR3244070-7), type II collagen (COL2A1; 1:50; Sigma-Aldrich; MAB8887; Lot#3215502), Aggrecan (1:100; Abcam; ab1031; Lot#3253326), and ADAMTS5 (1:50; GeneTex; gtx100332; Lot#39568)<br><br>Secondary antibodies used for immunohistochemistry were as follows;<br>donkey anti-rabbit IgG (H&L) conjugated with Biotin-SP (Jackson ImmunoResearch, 711-065-142; dilution 1:200), and goat-anti rat IgG (H&L) conjugated with Biotin (abcam, ab6844; dilution 1:200).<br><br>Primary antibodies used for western blotting were as follows;<br>OSCAR (PA5-47171; Lot#TK2675474; Thermo Fisher Scientific), MMP3 (Cat# Ab53015; Abcam), MMP13 (Cat# Ab39012; Abcam), ADAMTS5 (Cat# Ab441037; Abcam), COL2a1 (Cat# sc52658; Santa Cruz Biotechnology), ACAN (Cat# Ab3778; Abcam), SOX9 (Cat# 87630s; Cell Signaling Technology), p-Syk (Cat# 2711s; Cell Signaling Technology), Syk (Cat# 13198; Cell Signaling Technology), p-PLCγ2 (Cat# 3874S; Cell Signaling Technology), PLCγ2 (Cat# sc407; Santa Cruz Biotechnology), iNOS (Cat# Ab3523; Abcam; 1:1000 dilution), COX-2 (Cat# 12282; Cell Signaling Technology), LOX-5 (Cat# 3289; Cell Signaling Technology), p-p38 (Cat# 9211L, Cell Signaling Technology), p38 (Cat# 9212L, Cell Signaling Technology), p-JNK1/2 (Cat# 9251S, Cell Signaling Technology), JNK1/2 (Cat# 9252S, Cell Signaling Technology), p-ERK (Cat# 9101L, Cell Signaling Technology), ERK (Cat# 9102S, Cell Signaling Technology), p-IκBα (Cat# 9246S, Cell Signaling Technology), IκBα (Cat# 9242S, Cell Signaling Technology), p-p65 (Cat# 3031S, Cell Signaling Technology), p65 (Cat# 8242S, Cell Signaling Technology), p-Akt (Cat# 9271, Cell Signaling Technology), Akt (Cat# 9272, Cell Signaling Technology), β-actin (Cat# sc47778; Lot#C1919; Santa Cruz Biotechnology), GAPDH (Cat# sc32233; Lot#K3016; Santa Cruz Biotechnology)<br><br>Secondary antibodies used for western blotting were as follows;<br>goat anti-rabbit IgG (H&L) conjugated with HRP (Jackson ImmunoResearch, 111-035-003), goat anti-mouse IgG+IgM (H&L) conjugated with HRP (Jackson ImmunoResearch, 115-035-044), and donkey anti-goat IgG conjugated with HRP (Santa Cruz, sc-2020).<br><br>Primary antibodies used for immunofluorescence were as follows;<br>COX-2 (1:1000; Proteintech; Cat# 12375-1-AP; Lot#00093476). Normal mouse IgG is an affinity purified, unconjugated isotype control immunoglobulin from mouse<br><br>Secondary antibody used for immunofluorescence was as follows;<br>rabbit anti-mouse IgG+IgM (H&L) conjugated with Alexa Fluor 488 (Jackson ImmunoResearch, 315-485-044).
Validation	All antibodies used in this study were validated by the suppliers as follows;

Validation

Primary antibodies used for immunohistochemistry were as follows;
-OSCAR (1:200 dilution; Santa Cruz Biotechnology; D-19; sc34235; Lot#E2115) for IHC; species (Mouse, Human, Rat), application (WB, IF, ELISA)
manufacturer's website (https://datasheets.scbt.com/sc-34235.pdf)
-OSCAR (1:200 dilution; Biorbyt; orb185679; Lot#BR5396) for IHC; species (Mouse, Human, Rat), application (ELISA,ICC,IF,IHC-P)
manufacturer's website (https://www.biocompare.com/9776-Antibodies/5801021-OSCAR-antibody/)
-OSCAR (1:1000 dilution; Thermo Fisher Scientific; PA5-47171; Lot#TK2675474) for WB; species (Mouse), application (WB)
manufacturer's website (https://www.thermofisher.com/antibody/product/OSCAR-Antibody-Polyclonal/PA5-47171)
-MMP3 (1:50 dilution; Cat# Ab53015; Lot#GR3228789-8; Abcam;) for IHC; species (Mouse, Rat, Human), application (IHC-P, WB, ELISA, ICC/IF)
manufacturer's website (https://www.abcam.com/products/primary-antibodies/mmp3-antibody-ab53015.html)
-MMP13 (1:25 dilution;Cat# Ab51072; Lot#GR3244070-7; Abcam;) for IHC; species (Human), application (WB, Flow Cyt, ICC/IF, IHC-Fr, IHC-P or IP)
manufacturer's website (https://www.abcam.com/products/primary-antibodies/mmp13-antibody-ep1263y-ab51072.html)
-Aggrecan (1:100 dilution; Cat#3778; Lot#3253326; Abcam;) for IHC; species (Human, Mouse), application (IF, IHC-P, WB)
manufacturer's website (https://www.abcam.com/products/primary-antibodies/aggrecan-antibody-6-b-4-ab3778.html)
-COL2A1 (1:50 dilution; Cat# MAB8887; Lot#3215502; Sigma-Aldrich;) for IHC; species (Chicken, Human, Mouse, Salamander), application (IF, IHC, WB)
manufacturer's website (https://www.merckmillipore.com/KR/ko/product/Anti-Collagen-Type-II-Antibody-clone-6B3,MM_NF-MAB8887)
-ADAMTS5 (1:200 dilution; Cat# GTX100332; Genetex;) for IHC; species (Human, Mouse), application (WB, IHC-P)
manufacturer's website (https://www.genetex.com/Product/Detail/ADAMTS5-antibody/GTX100332)
-SOX9 (1:50 dilution; Cat# ab185966; Abcam; for IHC; species (Mouse, Human, Rat), application (Flow Cyt, ICC/IF, WB, IHC-P)
manufacturer's website (https://www.abcam.com/products/primary-antibodies/sox9-antibody-epr14335-78-ab185966.html)
-PPARγ (1:25 dilution; Cat# ab59256; Abcam;) for IHC; species (Human), application (WB, ICC/IF, IHC-P)
manufacturer's website (https://www.abcam.com/products/primary-antibodies/ppar-gamma-antibody-ab59256.html)
-GAPDH (1:1000 dilution; Santa Cruz Biotechnology; sc32233; Lot#K3016) for WB; species (Mouse, Rat, Human, Rabbit), application (WB, IHC, IF)
manufacturer's website (https://www.scbt.com/scbt/product/gapdh-antibody-6c5)
B-actin (1:1000 dilution; Santa Cruz Biotechnology; sc47778; Lot#C1919) for WB; species (mouse, rat, human, avian, bovine, canine, porcine, -rabbit, Dictyostelium discoideum, Physarum polycephalum), aplication (WB, IP, IF, IHC, ELISA)
manufacturer's website (https://www.scbt.com/p/beta-actin-antibody-c4)

Primary antibodies used for western blotting were as follows;
-OSCAR (1:1000 dilution; Cat# PA5-47171; Thermo Fisher Scientific)
manufacturer's website (https://www.thermofisher.com/antibody/product/OSCAR-Antibody-Polyclonal/PA5-47171)
-MMP3 (1:1000 dilution; Cat# Ab53015; Abcam)
manufacturer's website (https://www.abcam.com/products/primary-antibodies/mmp3-antibody-ab53015.html)
-MMP13 (1:1000 dilution; Cat# Ab39012; Abcam)
manufacturer's website (https://www.abcam.com/products/primary-antibodies/mmp13-antibody-ep1263y-ab51072.html)
-ADAMTS5 (1:1000 dilution; Cat# Ab41037; Abcam)
manufacturer's website (https://www.abcam.com/products/primary-antibodies/adamts5-antibody-ab41037.html)
-COL2a1 (1:1000 dilution; Cat# sc52658; Santa Cruz Biotechnology)
-ACAN (1:1000 dilution; Cat# Ab3778; Abcam)
manufacturer's website (https://www.abcam.com/products/primary-antibodies/aggrecan-antibody-6-b-4-ab3778.html)
-SOX9 (1:1000 dilution; Cat# 82630s; Cell Signaling Technology)
manufacturer's website (https://www.cellsignal.com/products/primary-antibodies/sox9-d8g8h-rabbit-mab/82630)
-p-Syk (1:1000 dilution; Cat# 2711s; Cell Signaling Technology)
manufacturer's website (https://www.cellsignal.com/products/primary-antibodies/phospho-syk-tyr525-526-antibody/2711)
-Syk (1:1000 dilution; Cat# 13198; Cell Signaling Technology)
manufacturer's website (https://www.cellsignal.com/products/primary-antibodies/syk-d3z1e-xp-rabbit-mab/13198)
-p-PLCγ2 (1:1000 dilution; Cat# 3874S; Cell Signaling Technology)
manufacturer's website (https://www.cellsignal.com/product/productDetail.jsp?productId=3874)
-PLCγ2 (1:1000 dilution; Cat# sc407; Santa Cruz Biotechnology)
manufacturer's website (https://www.scbt.com/ko/p/plc-gamma2-antibody-q-20)
-iNOS (1:1000 dilution; Cat# Ab178945; Abcam)
manufacturer's website (https://www.abcam.com/products/primary-antibodies/inos-antibody-epr16635-ab178945.html)
-COX-2 (1:1000 dilution; Cat# 12282; Cell Signaling Technology)
manufacturer's website (https://www.cellsignal.com/products/primary-antibodies/cox2-d5h5-xp-rabbit-mab/12282)
-LOX-5 (1:1000 dilution; Cat# 3289; Cell Signaling Technology)
manufacturer's website (https://www.cellsignal.com/products/primary-antibodies/5-lipoxygenase-c49g1-rabbit-mab/3289)
-p-p38 (1:1000 dilution; Cat# 9211L, Cell Signaling Technology)
manufacturer's website (https://www.cellsignal.com/products/primary-antibodies/phospho-p38-mapk-thr180-tyr182-antibody/9211)
-p38 (1:1000 dilution; Cat# 9212L, Cell Signaling Technology)
manufacturer's website (https://www.cellsignal.com/products/primary-antibodies/p38-mapk-antibody/9212)
-p-JNK1/2 (1:1000 dilutionCat# 9251S, Cell Signaling Technology)
manufacturer's website (https://www.cellsignal.com/products/primary-antibodies/phospho-sapk-jnk-thr183-tyr185-antibody/9251)
-JNK1/2 (1:1000 dilution; Cat# 9252S, Cell Signaling Technology)
manufacturer's website (https://www.cellsignal.com/products/primary-antibodies/sapk-jnk-antibody/9252)
-p-ERK (1:1000 dilution; Cat# 9101L, Cell Signaling Technology)
manufacturer's website (https://www.cellsignal.com/products/primary-antibodies/phospho-p44-42-mapk-erk1-2-thr202-tyr204-antibody/9101)
-ERK (1:1000 dilution; Cat# 9102S, Cell Signaling Technology)
manufacturer's website (https://www.cellsignal.com/products/primary-antibodies/p44-42-mapk-erk1-2-antibody/9102)
-p-IκBα (1:1000 dilution; Cat# 9246S, Cell Signaling Technology)

manufacturer's website (https://www.cellsignal.com/products/primary-antibodies/phospho-ikba-ser32-36-5a5-mouse-mab/9246)
-IκBα (1:1000 dilution; Cat# 9242S, Cell Signaling Technology)
manufacturer's website (https://www.cellsignal.com/products/primary-antibodies/ikba-antibody/9242)
-p-p65 (1:1000 dilution; Cat# 3031S, Cell Signaling Technology)
manufacturer's website (https://www.cellsignal.com/products/primary-antibodies/phospho-nf-kb-p65-ser536-antibody/3031)
-p65 (1:1000 dilution; Cat# 8242S, Cell Signaling Technology)
manufacturer's website (https://www.cellsignal.com/products/primary-antibodies/nf-kb-p65-d14e12-xp-rabbit-mab/8242)
-p-Akt (1:1000 dilution; Cat# 9271, Cell Signaling Technology)
manufacturer's website (https://www.cellsignal.com/products/primary-antibodies/phospho-akt-ser473-antibody/9271)
-Akt (1:1000 dilution; Cat# 9272, Cell Signaling Technology)
manufacturer's website (https://www.cellsignal.com/products/primary-antibodies/akt-antibody/9272)
-β-actin (1:1000 dilution; Cat# sc47778; Santa Cruz Biotechnology)
manufacturer's website (https://www.scbt.com/p/beta-actin-antibody-c4?requestFrom=search)
-GAPDH (1:1000 dilution; Cat# sc32233; Santa Cruz Biotechnology)
manufacturer's website (https://www.scbt.com/p/gapdh-antibody-6c5?requestFrom=search)

Primary antibodies used for immunofluorescence were as follows;
-COX-2 (1:1000 dilution; Cat# 12375-1-AP; Lot#00093476; Proteintech) for IF; species specificity (Human, Mouse, Rat), application (WB, IHC, IF)
manufacturer's website (https://www.ptglab.com/products/PTGS2-Antibody-12375-1-AP.htm)

Eukaryotic cell lines

Policy information about cell lines and Sex and Gender in Research

Cell line source(s)
The HEK293F cell line used in this study was obtained from Thermo Fisher Scientific (Cat No. R79007). This cell line is derived from human embryonic kidney cells and is widely recognized for its robust growth and high transfection efficiency.

Authentication
None of these cell lines were authenticated by us.

Mycoplasma contamination
The cell line was tested monthly to be negative for mycoplasma contamination.

Commonly misidentified lines
(See ICLAC register)
There was no commonly misidentified cell lines that used in our research.

Animals and other research organisms

Policy information about studies involving animals; ARRIVE guidelines recommended for reporting animal research, and Sex and Gender in Research

Laboratory animals
The study involved the following laboratory animals:
C57BL/6J male mice (purchased from Jackson laboratory and bred in the unit)
OSCAR knock out mice (embryos were a gift from Prof Yongwon Choi and mice were bred in house).
All mice used in experimental studies were males aged 8-12 weeks old weighing between 20-25g.
Experimental OA was induced by DMM (destabilization of the medial meniscus) surgery or IA (intra-articular) knee injection.
10-12 week old male mice were used.
For primary cell culture, chondrocytes were isolated from the femoral condyles and tibial plateaus of postnatal day 5 WT mice.

Wild animals
This study did not involve wild animals.

Reporting on sex
DMM : C57BL/6J WT male mice
Primary chondrocyte: neonatal 4-5-day-old ICR male and female mice
Detailed information can be found in the methods section.

Field-collected samples
This study did not involve animals collected from the field.

Ethics oversight
All animal experiments were approved by the institutional committee and conformed to the guidelines and laws set by the Laboratory Animal Genomic Center, Ewha, South Korea.

Note that full information on the approval of the study protocol must also be provided in the manuscript.

Plants

Seed stocks	*Report on the source of all seed stocks or other plant material used. If applicable, state the seed stock centre and catalogue number. If plant specimens were collected from the field, describe the collection location, date and sampling procedures.*
Novel plant genotypes	*Describe the methods by which all novel plant genotypes were produced. This includes those generated by transgenic approaches, gene editing, chemical/radiation-based mutagenesis and hybridization. For transgenic lines, describe the transformation method, the number of independent lines analyzed and the generation upon which experiments were performed. For gene-edited lines, describe the editor used, the endogenous sequence targeted for editing, the targeting guide RNA sequence (if applicable) and how the editor was applied.*
Authentication	*Describe any authentication procedures for each seed stock used or novel genotype generated. Describe any experiments used to assess the effect of a mutation and, where applicable, how potential secondary effects (e.g. second site T-DNA insertions, mosiacism, off-target gene editing) were examined.*
